# Comparison of the effects on coagulation function and safety of bivalirudin and heparin in patients undergoing percutaneous coronary intervention: A randomized trial

**DOI:** 10.1097/MD.0000000000040731

**Published:** 2024-11-29

**Authors:** Yanan Wang, Xiaorong Ren, Zhizhou Song, Qi Wu, Youdong Yang

**Affiliations:** a Department of Cardiovascular Medicine, Datong Third People’s Hospital, Datong, China; b Department of General practice, Datong Third People’s Hospital, Datong, China.

**Keywords:** bivalirudin, coagulation function, heparin, percutaneous coronary intervention, safety

## Abstract

**Background:**

To analyze the effects on coagulation function and safety of bivalirudin and heparin in patients undergoing percutaneous coronary intervention (PCI) and provide clinical evidence for their application.

**Methods:**

A total of 42 patients with coronary heart disease undergoing PCI treatment from July 2019 to January 2022 at Datong Third People’s Hospital in China were divided into 2 groups: the bivalirudin group and the heparin group. The former received perioperative administration of bivalirudin, while the latter received heparin. After 24 hours of treatment, blood indicators, coagulation functions, as well as cardiac, hepatic, and renal markers were evaluated. Additionally, Thrombolysis In Myocardial Infarction (TIMI) flow graded infarct-related vessel blood flow was assessed in both groups. Adverse cardiovascular and cerebrovascular events were monitored for a duration of 12 months.

**Results:**

The Activated clotting time (ACT), D-dimer (D-D), and prothrombin time (PT) levels in the bivalirudin group were significantly lower than those in the heparin group (*P* < .05). Both the bivalirudin and heparin groups showed significant improvement in TIMI flow grade after PCI (*P* < .05). The levels of Creatine Kinase-MB (CK-MB), N-terminal Pro-B-type Natriuretic Peptide (NT-proBNP) in the bivalirudin group were significantly lower than those in the heparin group (*P* < .05). There were no serious adverse cardiovascular and cerebrovascular events in either group.

**Conclusion:**

Bivalirudin has a slightly superior impact on coagulation function and safety profile in patients undergoing PCI compared to heparin, and the preventive effect of both on postoperative cardiovascular events is similar.

## 1. Introduction

Coronary artery disease is a common cardiovascular disorder, with 1 of its primary clinical manifestations being myocardial ischemia due to coronary artery narrowing, which in severe cases can lead to myocardial infarction.^[1]^ Percutaneous Coronary Intervention (PCI) is a significant therapeutic approach, playing a crucial role in alleviating symptoms of coronary artery disease, improving the quality of life, and reducing the incidence of cardiovascular events. However, PCI involves intravascular procedures, which can lead to abnormalities in coagulation function, increasing the risks of intraoperative and postoperative bleeding.^[2]^ Therefore, the management of coagulation function during the procedure becomes a critical concern during preoperative preparation and the surgical process. Currently, commonly used anticoagulant therapies in PCI include bivalirudin and heparin. Bivalirudin is a direct thrombin inhibitor that blocks thrombus formation by inhibiting the generation of thrombin.^[3]^ Heparin is an indirect thrombin inhibitor that enhances the activity of antithrombin (antithrombin III) to suppress thrombin formation.^[4]^ However, despite the widespread use of anticoagulants such as bivalirudin and heparin in PCI, their efficacy, safety, and differential impacts on coagulation function and safety in intraoperative patients have not been fully compared and evaluated. Therefore, this study aims to analyze and compare the effects and safety of bivalirudin and heparin on coagulation function in patients undergoing intraoperative PCI, and thus provide evidence for clinical applications.

## 2. Methods

### 2.1. Patient selection

In accordance with the approval of Datong Third People’s Hospital’s Ethics Committee (Approval no. 201809), a total of 42 patients with coronary heart disease who underwent PCI treatment between July 2019 and January 2022 were selected as the research subjects. All patients were divided into 2 groups based on different anticoagulant medications: the bivalirudin group, which included 27 cases and the heparin group, which included 15 cases. The declaration of Helsinki is otherwise followed.

### 2.2. Patient inclusion and exclusion criteria

#### 2.2.1. Inclusion criteria

1. Confirmed diagnosis of coronary heart disease with concurrent atrial fibrillation;
2. Meeting the indications for PCI and undergoing first-time PCI treatment;
3. No history of allergy to anticoagulant or antiplatelet drugs used in this study;
4. Voluntary participation in the study and signing of informed consent.

#### 2.2.2. Exclusion criteria

1. Severe systemic diseases affecting the brain, liver, kidneys, etc;
2. Severe bleeding, thrombocytopenia, immunodeficiency diseases, or severe coagulation disorders;
3. Concomitant diseases requiring long-term use of anticoagulant or antiplatelet aggregation drugs;
4. Malignant tumors;
5. History or echocardiography evidence of valvular heart disease, previous valve replacement surgery, dilated cardiomyopathy, restrictive cardiomyopathy, or hypertrophic cardiomyopathy;
6. Complex coronary artery lesions;
7. Underwent major surgical procedures within 30 days before enrollment;
8. Hemodynamically unstable patients, such as those in cardiogenic shock.

### 2.3. Treatments

All patients were given aspirin 100 mg/day for 3 days, and received a loading dose of clopidogrel 300 mg at least 6 hours before the procedure. In the bivalirudin group, a preoperative intravenous injection of bivalirudin (Shenzhen Xilantai Pharmaceutical Co., Ltd., H20110095, Shenzhen, China) at a dose of 0.75 mg/kg was administered before PCI, followed by a continuous intravenous infusion of 1.75 mg/(kg·h) during the procedure. In the heparin group, preoperative intravenous heparin (Beijing Saisheeng Pharmaceutical Co., Ltd., H11020362, Beijing, China) at a dose of 75 U/kg was administered, with an additional 1000 U/kg added for every hour exceeding 1 hour of procedure time. Blood samples were collected from all patients before, 8 hours and 24 hours after the intervention. CK-MB (Creatine Kinase-MB) and troponin I levels were measured. All patients were treated with aspirin 100 mg/day and clopidogrel 75 mg/day for at least 1 month after PCI.^[3]^

### 2.4. Observational measures

Fasting venous blood samples were collected from both groups of patients 24 hours after treatment. White blood cell (WBC), red blood cell (RBC), hemoglobin (HGB), and hematocrit (HCT) levels were measured to assess differences in blood routine indicators. Platelet (PLT), activated clotting time (ACT), activated partial thromboplastin time (APTT), D-dimer (D-D), thrombin time (TT), prothrombin time (PT), and fibrinogen (FIB) levels were monitored to evaluate coagulation function indicators. The Thrombolysis In Myocardial Infarction (TIMI) flow grade was used to assess the forward blood flow grading of infarct-related vessels. Alanine aminotransferase (ALT), aspartate aminotransferase (AST) levels were measured to evaluate hepatic function. Serum creatinine (Scr), blood urea nitrogen (BUN) and creatinine clearance rate (Ccr) levels were measured to evaluate renal function while cardiac troponin I (cTnI), CK-MB, and N-terminal Pro-B-type natriuretic peptide (NT-proBNP) levels were measured to evaluate cardiac function. Additionally, patients were followed up for at least 12 months to record the occurrence of adverse cardiovascular and cerebrovascular events, such as myocardial infarction, ischemic stroke, stent thrombosis, target vessel revascularization, and death.

### 2.5. Statistical analysis

Statistical analysis was conducted using Statistic Package for Social Science (SPSS) 20.0 (IBM, Armonk, NY, USA). Count data were presented as percentages and analyzed using the chi-square test for intergroup differences. Rank-sum tests were used for ordinal data differences. Measurement data were presented as mean ± standard deviation and analyzed using the LSD-*t* test for intergroup differences. A significance level of *P* < .05 was considered statistically significant.

## 3. Results

### 3.1. General patient characteristics

The bivalirudin group included 27 cases (21 males and 6 females), with an age range of 28 to 77 (61.70 ± 12.35) years, height range of 155 to 183 (168.33 ± 6.16) cm, weight range of 50 to 100 (69.19 ± 12.06) kg, and 14 smokers and 10 alcohol drinkers; while the heparin group, included 15 cases (11 males and 4 females), with an age range of 41 to 84 (63.27 ± 11.29) years, height range of 155 to 182 (169.27 ± 8.99) cm, weight range of 40 to 86 (68.27 ± 12.08) kg, and 9 smokers and 7 alcohol drinkers. There were no significant differences in general patient characteristics between the 2 groups (*P *> .05).

### 3.2. Patient blood routine indicator measurements

The results of this study showed no significant differences in WBC, RBC, HGB, and HCT levels between the bivalirudin group and the heparin group (*P* > .05), as shown in Table 1.

**Table 1:** Patient blood routine indicator measurements.

Indicators	Bivalirudin group	Heparin group	*t*	*P*
WBC (10^9^/L)	10.00 ± 2.63	9.93 ± 4.74	0.048	.962
RBC (10^12^/L)	4.79 ± 0.66	4.94 ± 0.49	0.529	.603
HGB (g/L)	147.11 ± 17.34	146.07 ± 13.05	0.406	.686
HCT (vol%)	43.76 ± 4.74	44.48 ± 3.64	0.616	.540

HCT = hematocrit, HGB = hemoglobin, RBC = red blood cell count, WBC = white blood cell count.

### 3.3. Coagulation function indicator measurements

The results of this study showed no significant differences in PLT, APTT, TT, and FIB levels between the bivalirudin group and the heparin group (*P* > .05). However, in the bivalirudin group, ACT, D-D, and PT levels were significantly lower than those in the heparin group (*P* < .05), as shown in Table 2.

**Table 2:** Coagulation function indicator measurements.

Indicators	Bivalirudin group	Heparin group	*t*	*P*
PLT (10^9^/L)	217.19 ± 70.38	228.27 ± 80.58	1.143	.254
ACT (s)	206.19 ± 39.85	237.94 ± 30.21	6.730	.000
APTT (s)	29.37 ± 4.17	31.07 ± 4.85	1.268	.211
D-D (mg/L)	0.20 ± 0.08	0.36 ± 0.10	6.347	.000
TT (s)	11.64 ± 0.84	12.03 ± 0.47	1.929	.061
PT (s)	11.57 ± 0.43	12.03 ± 0.39	2.767	.010
FIB (g/L)	2.54 ± 0.48	2.49 ± 0.65	0.183	.857

ACT = activated clotting time, APTT = activated partial thromboplastin time, D-D = D-dimer, FIB = fibrinogen level, PLT = platelet count, PT = prothrombin time, TT = thrombin time.

### 3.4. TIMI flow grade investigation

The results of this study demonstrated that both the bivalirudin and heparin groups showed significant improvement in TIMI flow grade after PCI (*P* < .05) compared to before PCI. There were no significant differences in TIMI flow grade between the 2 groups after PCI (*P* > .05), as shown in Table 3.

**Table 3:** TIMI flow grade investigation.

Groups	Time	0 to 1 Grade	2 Grade	3 Grade
Bivalirudin	Before PCI	25	0	2
	After PCI	1	0	26
U			6.466	
P			0.000	
Heparin	Before PCI	11	2	2
	After PCI	0	0	15
U			4.587	
P			0.000	
U_After PCI_			0.696	
P_After PCI_			0.487	

P = plavix, U = unfractionated heparin.

### 3.5. Cardiac, hepatic, and renal biomarker measurements

The results showed that there were no significant differences in ALT, Scr, BUN, Ccr, and cTnI levels between the bivalirudin group and the heparin group (*P* > .05). However, in the bivalirudin group, AST, CK-MB, and NT-proBNP levels were significantly lower than those in the heparin group (*P *< .05), as shown in Table 4.

**Table 4:** Cardiac, hepatic and renal biomarker measurements for both groups of patients.

Indicators	Bivalirudin group	Heparin group	*t*	*P*
ALT (U/L)	50.96 ± 28.10	64.34 ± 34.56	1.905	.060
AST (U/L)	167.08 ± 159.87	257.67 ± 185.68	4.329	.000
Scr (umol/L)	69.36 ± 27.60	67.32 ± 18.48	0.364	.717
BUN (mmol/L)	5.82 ± 1.81	5.78 ± 1.46	0.052	.959
Ccr (mL/min)	75.40 ± 46.06	62.88 ± 29.56	1.313	.193
cTnI (ng/mL)	3.58 ± 11.61	7.91 ± 19.02	0.680	.504
CK-MB (ng/mL)	22.47 ± 52.47	106.80 ± 136.20	2.305	.035
NT-proBNP (pg/mL)	99.50 ± 175.01	307.67 ± 140.74	11.189	.000

ALT = alanine aminotransferase, AST = aspartate aminotransferase, BUN = blood urea nitrogen, Ccr = creatinine clearance, CK-MB = Creatine Kinase-MB, cTnI = cardiac troponin I, NT-proBNP = N-terminal Pro-B-type natriuretic peptide, Scr = serum creatinine.

### 3.6. Investigation of adverse cardiovascular and cerebrovascular events in patients

In this study, 1 case in the bivalirudin group underwent stent implantation in the circumflex branch during PCI, and 1 case experienced chest tightness and shortness of breath. In the heparin group, 1 case reported back pain 1 year after the procedure. There were no other adverse cardiovascular and cerebrovascular events in both groups. Furthermore, all adverse cardiovascular evevnts were effectively relieved and treated with appropriate measures.

## 4. Discussion

PCI is a commonly used interventional procedure for treating patients with coronary heart disease.^[5]^ Its primary goal is to restore blood flow to the coronary arteries, improve cardiac blood supply, alleviate myocardial ischemia and pain, and prevent cardiac events (Myocardial Infarction, Angina, Arrhythmias, Heart Failure). The success of PCI directly impacts patient prognosis. Anticoagulant therapy plays a crucial role in PCI procedures. During PCI, the insertion of a stent (interventional device) into the coronary artery is often necessary to ensure its patency. However, this procedure can lead to endothelial damage within the blood vessels, potentially triggering platelet aggregation and coagulation reactions, resulting in thrombus formation.^[6]^ Anticoagulant therapy effectively inhibits platelet aggregation and coagulation factor activation, thus preventing intravascular restenosis and thrombus formation, thereby maintaining coronary artery patency. PCI procedures may lead to complications such as bleeding, particularly when patients are using antiplatelet drugs (such as aspirin). Anticoagulant therapy can partially control the coagulation state, reduce bleeding risks, and ensure the safety of the procedure.^[7]^ Appropriate anticoagulant therapy ensures the durability of stent implantation, reduces the risk of vascular restenosis, enhances the long-term success rate of the procedure, and improves patients’ quality of life.^[8]^

Bivalirudin is a direct thrombin inhibitor and belongs to the class of anticoagulants.^[9]^ Its main mechanism of action involves inhibiting the conversion of prothrombin to thrombin, thereby reducing the formation of blood clots. Bivalirudin’s anticoagulant effect is relatively predictable, allowing for more accurate control of a patient’s coagulation status and a reduction in the risks of bleeding and thrombosis.^[10]^ When used, bivalirudin may contribute to reduced platelet activation and decreased coagulation factor activation, which could help mitigate the risk of thrombus formation during the PCI procedure.

Heparin is a commonly used indirect thrombin inhibitor that functions by activating antithrombin III to inhibit the coagulation process.^[11]^ In PCI procedures, heparin has been a mainstream choice for a certain period of time. Heparin-based anticoagulants are an essential component of modern medicine.^[12]^ The anticoagulant effect of heparin can be reversed using agents such as protamine sulfate, which can be advantageous in cases of urgent situations like bleeding. Both bivalirudin and heparin have their unique advantages and suitable scenarios in the context of PCI procedures. Physicians typically choose the appropriate anticoagulant based on specific patient factors, including coagulation status, bleeding risk, and procedural characteristics.^[13]^

The BRIGHT 4 trial revealed that in patients with ST-elevation myocardial infarction (STEMI) undergoing primary PCI primarily via radial artery access, the use of bivalirudin along with a high-dose infusion for 2 to 4 hours after PCI significantly reduced the composite rate of all-cause mortality or major bleeding at 30 days when compared to heparin monotherap.^[14]^ Currently, there remains a relative lack of direct comparative studies between bivalirudin and heparin regarding their impact on coagulation function and safety in PCI procedures.^[15]^ With the ongoing advancement of medical technology and the potential influence of patient variability and procedural factors on the efficacy and safety of anticoagulant therapy. And by comparing the effects on coagulation function and safety of bivalirudin and heparin in PCI procedures, clinicians can be provided with more scientifically grounded guidance when selecting anticoagulant treatment regimens.^[14]^ This, in turn, can help reduce the risk of procedure-related complications and enhance the success rate of the procedures.

The study indicated that there were no significant differences between the bivalirudin group and the heparin group in terms of WBC, RBC, HGB, HCT, PLT, APTT, TT, and FIB levels. However, patients in the bivalirudin group exhibited significantly lower levels of ACT, D-D, and PT compared to the heparin group. This could be attributed to the fact that bivalirudin, being a direct thrombin inhibitor, can more precisely regulate the coagulation system, thereby reducing coagulation activity and affecting indicators such as ACT, D-D, and PT. Bivalirudin is an inhibitor of thrombin, a factor within the coagulation cascade essential to thrombus formation. Thrombin serves to cleave fibrinogen into fibrin. Fibrin monomers then convert factor XIII to factor XIIIa, allowing for the stabilization of the thrombus. Additionally, fibrin activates factor V and factor VIII, further promoting thrombin and platelet activation.^[16]^ TTR in patients using warfarin is 1 of the basic requirements for the efectiveness of treatment, studies have shown magnesium levels are an infuential factor in stabilizing INR.^[17]^ This provides us with a new idea, there may be some factors involved in bivalirudin’s regulation of coagulation system, Further research is still needed. The levels of cardiac enzyme markers (CK-MB, NT-proBNP) in the bivalirudin group were significantly lower than those in the heparin group, which might be linked to the impact of bivalirudin on the coagulation system and its reduced platelet activation. Lower levels of cardiac enzyme markers could reflect reduced myocardial injury, contributing to a decreased risk of postoperative cardiac events.

The study revealed that both the bivalirudin and heparin groups exhibited significant improvements in TIMI flow grade after PCI, with no significant differences between the 2 groups seen after PCI. This suggests that both bivalirudin and heparin effectively improve coronary blood flow, thereby aiding in ensuring myocardial perfusion. There were no significant differences in postoperative cardiovascular events between the 2 groups, and all adverse cardiovascular events were effectively relieved and treated. This outcome may be attributed to the effectiveness of both anticoagulant therapy and timely interventions for cardiac events. The analysis suggests that bivalirudin and heparin have distinct impacts in PCI procedures. Physicians should select the appropriate anticoagulant and dosage based on specific patient factors, including coagulation status, bleeding risk, and cardiovascular event risk, to maximize the success of the procedure and patient safety. The lower levels of cardiac enzyme markers in the bivalirudin group may reflect reduced myocardial injury, prompting clinical practitioners to take measures during the procedure to protect the myocardium and reduce the risk of postoperative cardiac events. Timely intervention for adverse cardiovascular events can effectively alleviate symptoms and reduce damage. As a result, physicians are reminded to closely monitor changes in patients’ conditions after the procedure and implement timely interventions to improve patient prognosis. This study provides valuable insights into anticoagulant therapy for clinical PCI procedures, emphasizing the significance of personalized treatment, myocardial protection, and postoperative monitoring.

## 5. Limitation of study

Nevertheless, it is important to note that this study may have limitations related to sample size, study design, and other factors. Therefore, careful consideration should be given when interpreting the results, and a comprehensive assessment should be made in conjunction with additional clinical evidence and guidelines.

## 6. Conclusion

In the context of PCI procedures, anticoagulant therapies with bivalirudin and heparin demonstrate differences in blood parameters, coagulation function, and cardiac enzyme markers with bivalirudin showing slight superiority. However, they both exhibited similar preventive effects on postoperative cardiovascular events. This underscores the importance of individualized treatment and myocardial protection. These findings can guide clinical practice, enhance the success rate of procedures, and improve patient prognosis.

## Author contributions

**Data curation:** Qi Wu.

**Formal analysis:** Xiaorong Ren.

**Investigation:** Zhizhou Song.

**Methodology:** Youdong Yang.

**Validation:** Yanan Wang.

**Writing – original draft:** Yanan Wang.

**Writing – review & editing:** Xiaorong Ren.
